# Exploring transcriptome plasticity at the intersection of cell signaling, metabolism and computational biology

**DOI:** 10.1038/s12276-025-01513-1

**Published:** 2025-08-14

**Authors:** Jeongsik Yong

**Affiliations:** Department of Biochemistry, Molecular Biology and Biophysics, University of Minnesota Twin Cities, Minneapolis, MN USA

**Keywords:** Alternative splicing, Long non-coding RNAs

Over the past decade, our understanding of the transcriptome has deepened notably, revealing unprecedented layers of complexity and regulation beyond the canonical view of gene expression. Far from being a static intermediary between DNA and proteins, the transcriptome is now recognized as a dynamic and responsive entity, shaped by posttranscriptional modifications, environmental cues, metabolic states and signaling networks. This special review section, ‘Advances in transcriptomics: cell signaling, metabolism, RNA regulation and computational insights’, brings together four timely and comprehensive articles that explore distinct yet interconnected dimensions of transcriptome plasticity.

## mTOR as a master regulator of RNA processing

The review by Yong et al. explores a rapidly emerging frontier in transcriptome biology: how the mechanistic target of rapamycin (mTOR) pathway governs RNA processing events such as alternative splicing and polyadenylation. Long known for its role in regulating translation and cell growth^1^, mTOR is now understood to extend its influence deep into the transcriptome. The authors provide a detailed examination of how mTOR signaling modulates transcriptome plasticity, affecting the isoform output of the genome and potentially altering protein localization, stability and interaction networks.

What distinguishes this review is its emphasis on context-dependent transcript remodeling. mTOR-mediated shifts in isoform expression are shown to contribute to adaptive responses and disease phenotypes—including tumor heterogeneity and treatment resistance^2–6^. This review also proposes that future work must move beyond transcript quantification to functional interrogation of isoform-specific roles, particularly in mTOR-driven disease contexts.

## Uridine as a metabolic and regulatory hub in cancer

In a complementary metabolic perspective, Kim et al. delve into the multifaceted biology of uridine—a ubiquitous ribonucleotide that serves not only as a building block for RNA but also as a metabolic currency in cancer cells. This review frames uridine metabolism at the intersection of nucleotide synthesis, glycolysis and mitochondrial function, emphasizing its role in both normal physiology and tumorigenesis. Importantly, it highlights how RNA decay and salvage pathways contribute to uridine pools, positioning RNA itself as a recyclable metabolic resource^7^.

The authors present compelling evidence that cancer cells exploit uridine metabolism under nutrient-limiting conditions, such as glucose starvation, suggesting that targeting this pathway could disrupt a key survival mechanism. Moreover, uridine’s role in modulating chemotherapy toxicity, ferroptosis and signaling via RNA-derived intermediates reveals novel therapeutic angles^8,9^. This review effectively bridges RNA biology with central metabolic regulation and opens the door to novel strategies targeting nucleotide metabolism in cancer.

## lncRNAs as regulators of enzymatic function in cancer

Yoon et al. expand the transcriptomic landscape by focusing on long noncoding RNAs (lncRNAs) as critical regulators of cancer cell metabolism and enzymatic activity^10,11^. This review shifts the attention from coding mRNAs to the expansive noncoding transcriptome, spotlighting lncRNAs that directly interact with metabolic enzymes and modulate posttranslational modifications.

The authors classify lncRNAs on the basis of their mechanisms of action: scaffolding metabolic enzyme complexes, modifying enzymatic activity or influencing posttranslational modification machinery such as ubiquitination and acetylation. They detail several exemplary cases, including NEAT1_1, gLINC and HULC, which orchestrate metabolic rewiring in cancer by regulating glycolytic flux or protein stability^12–14^. This review underscores the emerging view that lncRNAs are not passive transcriptional byproducts but act as fine-tuners of cellular processes, deeply embedded in metabolic and signaling circuits. Importantly, it encourages deeper mechanistic studies to unravel how lncRNAs can be leveraged as therapeutic targets or biomarkers.

## Computational advances in alternative polyadenylation and alternative splicing analysis

Finally, Zhang et al. provide a comprehensive review of computational methodologies for analyzing alternative splicing and alternative polyadenylation—two essential mechanisms driving transcriptome plasticity. As the complexity of transcript isoform landscapes continues to grow, so does the demand for robust, scalable and accurate computational tools. This review categorizes methods for both bulk and single-cell RNA sequencing data, providing a valuable resource for researchers navigating the expanding toolkit for isoform discovery and quantification.

A unique strength of this article is its coverage of intronic polyadenylation, a subtype of alternative polyadenylation that generates truncated mRNAs with distinct functions. By highlighting recent deep learning and statistical models—such as PolyAMiner, DaPars and InPACT—the authors bridge computational innovation with biological insight^15–17^. The review also stresses the need for integrative approaches combining multi-omics data to decipher the functional impact of isoform changes, especially in disease contexts.

## Unifying themes and future directions

Despite covering different aspects of transcriptome regulation, these four reviews collectively emphasize several unifying themes:

1. Context-specific transcriptome remodeling is a hallmark of cellular adaptation, and dysregulation of this plasticity contributes to disease.
2. Transcript diversity matters—in both coding and noncoding RNAs—shaping protein function, metabolic flux and cellular signaling.
3. Computational frameworks are essential to decode the layers of RNA regulation, particularly in the age of high-throughput and single-cell technologies.
4. Interdisciplinary integration of cell signaling, metabolism, RNA biology and computational analysis is no longer optional—it is fundamental to understanding modern biology.

Looking forward, the challenge is not merely to identify new RNA isoforms but to understand their functional consequences in specific cellular and disease contexts. This will require (1) isoform-specific perturbation tools (for example, CRISPR base editors and antisense oligonucleotides), (2) proteogenomic approaches to map translation products of alternative isoforms and (3) in vivo models that capture tissue- and condition-specific isoform functions. Moreover, as shown in these reviews, transcriptomics is increasingly metabolically and enzymatically entangled—a revelation that may reshape our definitions of gene expression regulation altogether.

## Conclusion

The reviews in this special section highlight that RNA regulation is not a peripheral aspect of gene expression but a central node where signaling, metabolism and disease converge. Together, they offer a forward-looking perspective on transcriptome plasticity that is mechanistically rich, computationally grounded and biologically urgent. We hope this collection will stimulate new cross-disciplinary conversations, spark mechanistic discoveries and accelerate the development of RNA-targeted therapeutics.
